# The Nutritional Value and Biological Activity of Concentrated Protein Fraction of Potato Juice

**DOI:** 10.3390/nu11071523

**Published:** 2019-07-04

**Authors:** Przemysław Łukasz Kowalczewski, Anna Olejnik, Wojciech Białas, Iga Rybicka, Magdalena Zielińska-Dawidziak, Aleksander Siger, Piotr Kubiak, Grażyna Lewandowicz

**Affiliations:** 1 Institute of Food Technology of Plant Origin, Poznań University of Life Sciences, 60-624 Poznań, Poland; 2 Department of Biotechnology and Food Microbiology, Poznań University of Life Sciences, 60-627 Poznań, Poland; 3 Department of Technology and Instrumental Analysis, Poznań University of Economics and Business, 61-875 Poznań, Poland; 4 Department of Biochemistry and Food Analysis, Faculty of Food Science and Nutrition, Poznań University of Life Sciences, 60-623 Poznań, Poland

**Keywords:** potato juice, cancer cells, cytotoxicity, antioxidant activity, protein nutritional value, chemical score, protein composition

## Abstract

Potato protein is recognized as one of the most valuable nonanimal proteins due to the high content of essential amino acids. So far, it has not been used in human nutrition on a large scale due to technological limitations regarding its acquisition. In this study, the protein fraction of potato juice was concentrated with the use of membrane separation. The obtained potato juice protein concentrate (PJPC) was characterized in terms of nutritional value and biological activity, and the amino acid composition, mineral content, and antioxidant properties were determined. Moreover, in vitro cytotoxic activity against cancer cells of the gastrointestinal tract was investigated. The results of the present study indicate that PJPC is an excellent source of lysine and threonine, while leucine is its limiting amino acid, with an amino acid score (AAS) of 65%. Moreover, PJPC contains substantial amounts of Fe, Mn, K, and Cu. As demonstrated experimentally, PJPC is also characterized by higher antioxidant potential than potato itself. Biological activity, however, is not limited to antioxidant activity alone. Cytotoxicity studies using a gastric cancer cell line (Hs 746T), a colon cancer cell line (HT-29), and human colon normal cells (CCD 841 CoN) proved that PJPC is characterized by selective activity against cancer cells. It can thus be concluded that the developed method of producing protein concentrate from potato juice affords a product with moderate nutritional value and interesting biological activity.

## 1. Introduction

Potato juice, the liquid fraction of potato tubers, is available in large quantities as a side stream of the starch industry. Optimal management of this raw material is still an unresolved technological problem. The most common approach utilizes thermal coagulation of potato protein in acidic conditions. The coagulated mass is then separated, dried, and used for fodder purposes [1]. This procedure is not optimal as only ca. 50% of potato protein undergoes coagulation and often the remaining half is simply discarded with wastewater [2]. Moreover, not only proteins are lost but also many different compounds with proven biological activity [3,4,5]. *Solanum tuberosum* reveals vast biodiversity; approximately 5000 varieties of potato are known [6]. The content of the individual chemical components is determined by both the variety of the cultivated plant and the technological treatment of mature tubers [7,8,9]. The dry matter content varies between 20 and 50 g/kg, of which the weight of protein amounts to about one-third. Three main groups of proteins can be distinguished in potato: the major potato tuber protein patatin, with a molecular weight of approx. 39–45 kDa; protease inhibitors (4–25 kDa); and other proteins of higher molecular weight [10,11]. Moreover, except for the easily soluble protein fraction, potato juice also contains carbohydrates, lipids, organic acids, polyphenols, minerals, fiber, and glycoalkaloids.

Investigations into the nutritional value of potato proteins have resulted in a number of patent descriptions of new technologies for the production of food-grade potato protein [12,13,14,15], as well as scientific reports that concern its functional properties [16,17,18,19]. However, commercial applications of these results remain unsatisfactory. It should be emphasized that the value of potato juice as an industrial raw material is based not only on its nutritional value but also on its biological activity. In European folk medicine, the interest in freshly squeezed potato juice and its use for the treatment of gastrointestinal complaints can be traced back to the first decade of the 19th century [20,21]. Because of its documented anti-inflammatory activity within the gastrointestinal tract [22], potato juice can be used as an additive in the production of health-promoting foods given to patients suffering from inflammatory bowel diseases [23,24,25,26]. It is widely believed that the anti-inflammatory properties of potato are associated with proteins that manifest protease inhibitor activity. Published data indicate that potato proteins have the ability to relieve perirectal inflammation in patients with gastrointestinal resections, as well as in infants [27]. They may also be responsible for regulating the activity of endogenous proteases and performing protective functions against infections by pathogenic microorganisms or insects [28]. Furthermore, they have been shown to inhibit the activities of trypsin, chymotrypsin, cathepsin D, carbopeptidase A and B, and microbial proteases [29,30,31,32].

Potato protein intended for consumption must be subjected to thermal treatment, which may affect its biological activity or nutritional value. In vivo studies on the anti-inflammatory activity of spray-dried potato juice proved, however, that thermal treatment does not impact the biological activity [22]. Therefore, the aim of this study was to investigate the antioxidant activity, cytotoxicity, and nutritional composition of potato juice protein concentrate preserved by spray-drying. In addition, nutritional properties of protein and amino acid scoring were investigated with respect to FAO recommendations.

## 2. Materials and Methods

### 2.1. Experimental Material and Production of Protein Concentrate

**Figure 1:**
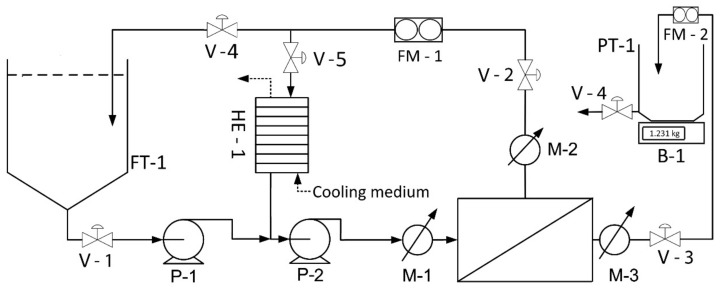
Schematic diagram of the applied membrane separation system for ultrafiltration. FT-1: feed tank; V-1–5: manual diaphragm valves; P-1, 2: centrifugal pumps; HE-1: shell and tube heat exchanger; M-1–3: pressure sensors; PT-1: permeate tank; FM-1, 2: electromagnetic flowmeters; B-1: electronic balance.

Fresh potato juice (FPJ) was collected during starch production season at “TRZEMESZNO” Sp. z o.o. Potato Industry Company (Trzemeszno, Poland). In starch processing, about 25 varieties of potato, with white and yellow flesh, were used, mainly: Albatros, Skawa, Dominanta, Harpun, and Jubilat. The material was subjected to concentration by means of cross-flow ultrafiltration (UF). A diagram of the setup used for this is presented in Figure 1. A polyethersulfone spiral-wound UF membrane (type 3838, Koch Membrane Systems, Inc., Wilmington, MA, USA) with a molecular weight cut-off of 5 kDa and an area of 3.5 m^2^ was used to separate proteins from the potato-processing wastewater. The experiments were carried out in an open system where the permeate was drained into a separate container (concentration mode). Concentration was performed at a transmembrane pressure (TMP) of 400 ± 15 kPa, cross-flow velocity inside the membrane of 0.5 ms^-1^, and temperature of 20 °C. The obtained retentate (potato juice protein concentrate, PJPC) was further subjected to spray-drying to ensure long-term storage stability.

Spray-drying was carried out in a Mobile Minor™ 2000 spray dryer (GEA Co., Søborg, Denmark) using the following conditions: 170 °C at the inlet to the drying chamber and 95 °C at the outlet.

### 2.2. Chemical Analysis

Total nitrogen content was determined using the Kjeldahl method according to ISO 1871 [33] and protein content was calculated by multiplying the result by the conversion factor of 6.25. Ash content was determined according to ISO 763 [34]. The α-chaconine and α-solanine concentrations were analyzed using high-performance liquid chromatography (HPLC; Waters, Milford, MA, USA) with an XBridge C18 column (3.5 μm, 3.0 x 100 mm) (Waters, Milford, MA, USA) with the temperature controlled at 70 °C. The injection volume was 10 µL. Isocratic separation was carried out at a flow rate of 1.0 mL/min. The mobile phase was a mixture of acetonitrile and 0.1 M KH_2_PO_4_ (20:80 v/v). Detection was carried out at a wavelength of 200 nm with a Waters 2998 detector (DAD) (Waters, Milford, MA, USA) [35].

### 2.3. Analysis of Amino Acid Composition and Nutritional Profile

#### 2.3.1. Amino Acid Composition

Amino acid composition, including histidine (His), isoleucine (Ile), leucine (Leu), lysine (Lys), methionine (Met), phenylalanine (Phe), threonine (Thr), valine (Val), cysteine (Cys), tyrosine (Tyr), glycine (Gly), arginine (Arg), proline (Pro), aspartic acid (Asp), glutamic acid (Glu), alanine (Ala), and serine (Ser), was determined with ultraperformance liquid chromatography (UPLC; Shimadzu Nexera 2.0, Kyoto, Japan) equipped with a PDA and FL detector (Kyoto, Japan) after acidic hydrolysis (110 °C, 23 h) according to the method described by Tomczak et al., 2018 [36], while sulfuric amino acids were prepared by oxidation (4 °C, 16 h) followed by acidic hydrolysis (110 °C, 2.5 h) [37]. The PDA detector was set at 260 nm with a sampling rate of 20 points/s. Tryptophan (Trp) content was determined after 20 h of alkaline hydrolysis and determined by reversed-phase UPLC/FD (excitation and emission at 280 and 356 nm, respectively) [38]. Amino acid content was expressed in g/16 g N (which is equivalent to g/100 g of protein).

#### 2.3.2. Protein Efficiency Ratio (PER)

The PER was calculated from the amino acid composition of PJPC based on the following three equations in accordance with the method described by Alsmeyer et al. [39]:

$$PER_{1}\;=\;-\;0.684\;+\;0.456\;\times \;Leu\;–\;0.047\;\times \;Pro$$

$$PER_{2}\;=\;-\;0.468\;+\;0.454\;\times \;Leu\;–\;0.105\;\times \;Tyr$$

$$PER_{3}\;=\;-\;1.816\;+\;0.435\;\times \;Met\;+0.78\times \;Leu\;+0.211\times \;His\;-\;0.944\times \;Tyr$$

#### 2.3.3. Scoring of Amino Acids

The amino acid score (AAS) was calculated for adults using the standard method recommended by the FAO [40]:

$$AAS=\;\frac{essential\;amino\;acids\;contents\;in\;PJPC\;\left[\%\right]}{recommended\;essential\;amino\;acids\;\left[\%\right]}.$$

#### 2.3.4. Digestibility of PJPC

The digestibility of the provided preparations was determined in vitro by a method simulating multienzymatic two-stage (gastric and intestinal) digestion [41]. The digestive phase in the oral cavity was omitted as it has no effect on the digestibility of protein preparations. The large intestine stage (where the release of amino acids is the result of microflora activity) was also not taken into account.

A sample was introduced into distilled water containing pepsin (60,000 U) (Sigma) and the pH of the mixture was lowered to 2.0 with 1 M HCl. Gastric digestion was carried out for 2 h at 37 °C. Then, the pH of the solution was adjusted to 7.4 and a solution containing pancreatic–intestine extract (0.005 g, Sigma) and bile salts (0.03 g, Sigma) in 5 mL 0.1 M NaHCO_3_ was added. The digestion was again performed at 37 °C for 2 h. The obtained solution was centrifuged and the undigested protein was precipitated with trichloroacetic acid. Protein nitrogen was determined using the Kjeldahl [33] method. A ratio of the result and the total amount of protein nitrogen introduced was used to calculate the digestibility.

### 2.4. Determination of Mineral Content

The concentrations of minerals Ca, Cu, Fe, K, Mg, Mn, Na, and Zn were determined using flame atomic absorption spectroscopy (F-AAS) (SpectrAA-800, Varian, Palo Alto, CA, USA) preceded by mineralization with nitric acid [42]. The percent of population reference intake (PRI) and adequate intake (AI) was calculated according to the latest EFSA recommendations [43]. Contents of minerals were expressed in g/100 g of the sample.

### 2.5. Antioxidant Activity and Total Phenolic Compound Content

#### 2.5.1. PJPC Ethanolic Extract Preparation

The extracts were prepared according to Król et al. [44]. PJPC (0.025 g) was diluted in 1.5 mL of 70% ethanol in an Eppendorf-type tube and sonicated for 45 min at 50 °C. Subsequently, the samples were centrifuged at 14,000 rpm for 10 min. Supernatants were used for further analysis. The extracts were prepared in six replicates.

#### 2.5.2. Determination of Antioxidant Activity

The antioxidant activity was assessed using the Trolox equivalent antioxidant capacity (TEAC) method against the ABTS radical (2,2′-azinobis-(3-ethylbenzothiazoline-6-sulfonic acid) [45]. Ferric-reducing antioxidant power (FRAP) was analyzed according to the procedure described by Benzie and Strain, 1996 [46]. Activity of PJPC was expressed as TEAC (in mmol Trolox g^-1^ of product) and FRAP (mmol of Fe^2+^ g^-1^) values.

#### 2.5.3. Determination of Total Phenolic Compounds (TPCs)

The TPC content was determined by the Folin–Ciocalteu colorimetric method [47]. For this, 0.02 mL of the sample was mixed with 0.05 mL of the Folin–Ciocalteu reagent. After 3 min, 0.15 mL of 20% sodium carbonate and 0.78 mL of demineralized water were added and the solution was mixed. The mixture was incubated in the dark at room temperature for 2 h. After 2 h, samples were centrifuged for 5 min at 14,000 rpm to obtain a clear solution. Absorbance was measured at 765 nm. The total content of phenolics was expressed in milligrams of gallic acid per gram of product.

#### 2.5.4. Determination of Total Flavonoid Compounds (TFCs)

The concentration of TFCs was determined according to Karadeniz et al. [48] (±)‑catechin was used as the standard. 0.12 mL of each sample was mixed with 0.60 mL of demineralized water and 0.036 mL of 5% NaNO_2_. After 5 min, 0.072 mL of 10% AlCl_3_ and 0.24 mL of 1 M NaOH were added. Following another 5 min incubation, 0.132 mL of demineralized water was added and the solution was mixed. Absorbance was measured at 510 nm. The total content of flavonoids was expressed in milligrams of (±)‑catechin per gram of product.

### 2.6. Cell Cultures

The human colorectal adenocarcinoma cell line HT-29 (Cat. no: 85061109) was obtained from the European Collection of Authenticated Cell Cultures (ECACC) and supplied by Sigma–Aldrich (Poznań, Poland). The human gastric carcinoma Hs 746T cell line (ATCC^®^ HTB-135^™^) and human normal colon CCD 841 CoN (ATCC^®^ CRL-1790^™^) cell line were obtained from the American Type Culture Collection (ATCC) and supplied by LGC Standards (Łomianki, Poland).

The HT-29 and Hs 746T cells were cultured in Dulbecco’s Modified Eagle’s Medium (DMEM; Sigma–Aldrich) supplemented with heat-inactivated fetal bovine serum (FBS; Gibco BRL, Grand Island, NY, USA) to a final concentration of 10% and 1% nonessential amino acids 100X (Sigma–Aldrich, Poznań, Poland). The base medium for CCD 841 CoN cells was ATCC-formulated Eagle’s Minimum Essential Medium with 10% FBS addition.

Cell cultures were incubated at 37 °C in a humidified atmosphere (5% CO_2_, 95% air).

### 2.7. In Vitro Cytotoxicity Assay

Cells were grown in 96-well plates at an initial density of 2.5 × 10^4^ cells/cm^2^. The 24 h cultures were treated with spray-dried potato juice at concentrations ranging from 0.5 to 20 mg/mL and incubated for 48 h under standard culture conditions. Cell viability and metabolic activity were determined using the 3-(4,5-dimethylthiazol-2-yl)-2,5-diphenyltetrazolium bromide (MTT) colorimetric assay [49] as described previously by Olejnik et al., 2016 [50]. The first cytotoxic dose (IC_10_), median effective concentration (IC_50_), and lethal dose (IC_90_) of the spray-dried potato juice protein concentrate were calculated on the basis of the MTT results. Cell response to PJPC treatment was evaluated concerning control cells not treated with PJPC.

### 2.8. Statistical Analysis

All measurements were repeated in triplicate, unless otherwise stated. One-way analysis of variance (ANOVA) was carried out independently for each dependent variable. A post hoc Tukey’s HSD multiple comparison test was used to identify statistically homogeneous subsets at α = 0.05. Statistical analysis was performed with Statistica 13 software (Dell Software Inc., Round Rock, TX, USA).

## 3. Results and Discussion

### 3.1. Nutritional Value of PJPC

The proximate compositions of FPJ and PJPC are presented in Table 1. Table 2 presents the determined amino acid score of the analyzed concentrate. Research has shown that potato proteins are of moderate nutritional value due to their amino acid composition. The chemical score of potato protein is up to 70, which makes it comparable to egg white [51,52,53]. PJPC can be used to enrich products in the limiting amino acids. Potato proteins are an excellent source of lysine and can thus be a good supplement to cereal products that contain low quantities of this amino acid [54,55]. The prepared PJPC (Table 2) had an AAS of 65, which is comparable to that of potato, but more importantly, only two of the essential amino acids were in amounts that did not provide the required supply. Leucine was the limiting amino acid of PJPC. The histidine content amounted to 86% of the egg standard value. The threonine content was much higher compared with the egg standard, which may exert a positive effect on the nitrogen balance in the body [56]. 

The nutritional value of a protein may be estimated by calculating the PER. This calculation is based on the concentration of either leucine and proline (PER_1_); leucine and tyrosine (PER_2_); or methionine, leucine, histidine, and tyrosine (PER_3_). Usually, PER values higher than 2 indicate the high quality of a protein. PER values calculated for PJPC indicated good quality of the protein (Table 3) that was higher than those of cowpea and peanut proteins (1.21 and 1.45–1.76, respectively) [57].

Currently, the nutritional value of a protein is also evaluated by a multienzyme digestion model. For the PJPC, a two-stage model was chosen, because the preparation is free of starches and the effect of salivary enzymes does not influence the final results of the experiment. The determined digestibility of PJPC was unexpectedly low (18.3%), significantly lower compared with reference data [58]. This was probably a result of the preparation method. Even in the case of valuable alimentary goods, such as almond protein, or popular food products, such as corn-based breakfast cereals, digestibility may be limited [59,60]. Increased temperature is known to decrease protein digestibility even though thermal denaturation by itself improves it. Thermal treatment was found to promote lysine modification, which significantly restricts its availability to trypsin [61]. Another disadvantage of spray-drying is its possible direct negative impact on the content of lysine [62]. To improve digestibility, it may be desirable to treat PJPC using proteases with low substrate specificity, such as bromelain, papain, or commercial preparations (e.g., Alcalase^®^ or Savinase^®^). It should, however, be taken into account that phenolic compounds are known inhibitors of proteases. A high concentration of these antioxidants may have had a strong effect on the digestibility of PJPC [63]. Noteworthy, in our previous investigations, potato juice and products of its processing were found to be rich in polyphenols and other antioxidants [35].

The contents and percentages of PRI and AI realization for minerals are shown in Table 4. The PRI and AI values were: 950 mg for Ca, 3500 mg for K, 300 mg for Mg, 1 mg for Cu, 16 mg for Fe, 3 mg for Mn, and 10 mg* for Zn (* the averaged value) [43].

PJPC was found to be a valuable source of the most of analyzed macro- and microelements, excluding Ca. Its 100 g portion realized 60–80% of daily requirements for Mg and Zn. PJPC was particularly rich in Fe, Mn, K, and Cu (approximately 190%, 130%, 120%, and 110% of PRI/AI, respectively). In terms of the content of minerals, PJPC was comparable to dietary supplements. As shown in our previous studies, potato juice in different forms is sensorially compatible with various food products [23,24]. Therefore, it may become a valuable ingredient for the enrichment of food. Furthermore, the gluten-free nature of PJPC makes it suitable for the enrichment of gluten-free food targeted at patients with gluten-related disorders. For example, the nutritional value of gluten-free bread is usually lower than that of bread made from conventional raw materials. Its production is often based mainly on pure starch. In addition, in order to ensure the right consistency and structure, large amounts of fat and sugar are added [64,65]. This significantly reduces the protein content. Moreover, gluten-free foods often contain an insufficient content of mineral compounds. Published data indicate that the gluten-free diet is deficient in Fe, Mg, and Zn [66,67]. The use of PJPC as an additive in the production of gluten-free bread could increase its protein content and help supply the deficient minerals. In this way, it could significantly improve the nutritional value of the product.

Caution must be paid to the content of toxic and antinutritional constituents of potential food additives of natural origin. In the case of potato and the products of its processing, two glycoalkaloids—α-solanine and α-chaconine—can pose a threat to potential consumers. In the case of the analyzed product, the content of any of these compounds was lower than 1 mg per 100 g of the product (Table 1). According to the available toxicological data, such concentrations should not create a significant risk [68,69], provided that PJPC is used as an additive in a limited quantity.

### 3.2. Antioxidant Activity of PJPC

Plant raw materials, such as fruits and vegetables, are well-recognized sources of antioxidant compounds [70,71,72]. Among antioxidant substances, phenols are characterized by the highest antioxidant activity [73,74,75]. Literature data indicate the anti-inflammatory effect of potatoes and attribute it to the presence of antioxidants, including phenolic acids, carotenoids, or anthocyanins. Human cohort studies have confirmed the systemic anti-inflammatory effect of potato and correlated it with the concentration of certain potato antioxidants in the blood serum [76,77,78]. The results of the analysis of antioxidant properties with respect to the content of dry matter (Table 5) showed that PJPC was characterized by a higher antioxidant potential than selected vegetables [79] or whole potato [80,81]. Moreover, its antioxidant activity was also approximately 10 times higher than fresh potato juice [35]. The content of polyphenolic compounds in PJPC was even two times higher than in flesh-colored potatoes that are rich in anthocyanin pigments and polyphenols [55]. Because of their capabilities to inhibit lipid oxidation, inactivate reactive oxygen species, or chelate prooxidant transition metals, proteins can be used as antioxidant additives in the production of food [82]. PJPC may thus not only be applied as an additive to increase the nutritional value of a product but also to introduce antioxidant properties to it.

### 3.3. In Vitro Cytotoxic Activity

The biological activity of potato juice is usually associated with the proteins present in it. Kujawska et al. [22] proved that potato juice inhibits the inflammatory response in experiments on activated macrophages in vitro and in vivo in the gastric mucosa with induced lesions in rats. Additionally, studies indicate that protease inhibitors present in the potato protein inhibit the fecal proteolytic activity almost completely. Therefore, the use of potato proteins can be a new approach in the prevention of protease-induced perianal dermatitis [27].

**Figure 2:**
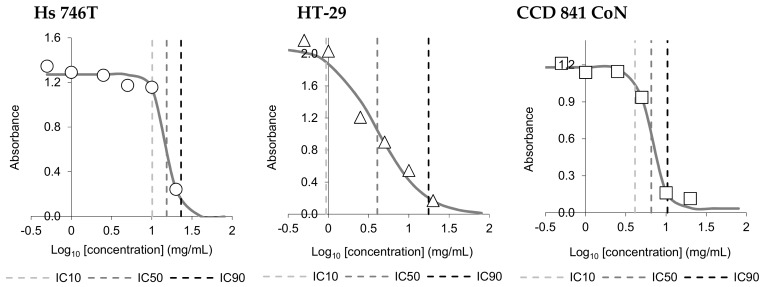
Changes in cell proliferation and viability upon treatment of the stomach cancer cells (Hs 746T line), colon cancer cells (HT-29 line), and colon normal cells (CCD 841 CoN line) with PJPC at concentrations ranging from 0.5 to 20 mg/mL.

The cytotoxicity of PJPC was determined using a gastric cancer cell line (Hs 746T), a colon cancer cell line (HT-29), and human colon normal cells (CCD 841 CoN). The IC_10_, IC_50_, and IC_90_ cytotoxic doses of PJPC are presented in Table 6. The effects on cell proliferation and viability in model tumor and nontumor cultures are shown in Figure 2. The results indicate differences in the cytotoxic activity of PJPC against cancer (HT-29) and normal (CCD 841 CoN) colon cells. This may be a fact of therapeutic significance, as commonly used anticancer drugs inhibit the proliferation of both cancerous and normal cells, which may lead to additional weakening of the body [83]. In the case of stomach tumor cells of the Hs476T line, the cytotoxic dose IC_50_ was relatively high. Direct comparison such as the one above was impossible, however, as normal cells from the human stomach were not available. Published data indicate cytotoxic activity of potato proteins towards various tumor lines [18,84]. The antiproliferative effects of potato could also be associated with the presence of glycoalkaloids (solanine and chaconine), which may inhibit the growth of human cancer cell lines, including human colon and stomach cancer cells [85,86,87]. Table 1 presents the content of glycoalkaloids in both fresh juice and PJPC. The developed method of obtaining PJPC caused a decrease in the content of glycoalkaloids compared with the raw material. Nonetheless, high cytotoxic activity, comparable to the published data for fresh potato juice, was maintained even after the treatment [35].

## 4. Conclusions

Potato juice, a low-value side-product of potato starch processing, is a valuable source of functional compounds with therapeutic potential. Valorization of this material is possible provided that sufficient knowledge of its properties is obtained through scientific and technological research. In its raw state, potato juice is unstable and must be processed in order to allow the necessary storage stability. PJPC, a product obtained from fresh potato juice by means of ultrafiltration which is stabilized by spray-drying, was the subject of the research presented herein. Analyses of the amino acid composition proved that it is a rich source of some essential amino acids, especially threonine. The determined AAS of PJPC was 65, with leucine as the limiting AA. Also interesting were the results on the mineral composition of PJPC. A 100 g portion of this material would supply Mn, K, and Cu in amounts surpassing the PRI/AI recommendation by approximately 20%, and its Fe content was especially high, with a 100 g portion supplying almost twice the recommended intake. The nutritional value of the product makes it a potential additive for the production of gluten-free bread, where it could be used to introduce both protein and minerals that are often deficient in the gluten-free diet. Besides chemical analyses, in vitro studies with the use of cancerous and normal cells from the human gastrointestinal tract were performed. These revealed differences in the potency of cytotoxicity of PJPC against normal (CCD 841 CoN) and cancerous (HT-29) colon cells that could potentially be exploited in therapeutic applications.

## Figures and Tables

**Table 1:** Proximate composition.

Parameter	FPJ	PJPC
Moisture (%)	94.49 ± 3.21 ^a^	4.79 ± 0.32 ^b^
Protein (g/100 g dm)	2.37 ± 0.07 ^b^	63.40 ± 0.82 ^a^
Ash (g/100 g dm)	0.91 ± 0.03 ^b^	7.54 ± 0.27 ^a^
α-chaconine (µg/100 g dm)	990.06 ± 17.11 ^a^	837.81 ± 15.36 ^b^
α-solanine (µg/100 g dm)	601.24 ± 19.37 ^a^	438.40 ± 18.49 ^b^

Mean values denoted by different letters (a, b) showed statistically significant difference (*p* < 0.05). FPJ—fresh potato juice; PJPC—potato juice protein concentrate.

**Table 2:** Amino acid profile and amino acid score (AAS) for adults according to standards reported by the FAO/WHO [40].

Amino Acid	FAO/WHO Standard (mg/g)	PJPC (g/16 g N)	AAS
Essential amino acids			
Histidine	16	1.95 ± 0.07	86.3
Isoleucine	30	4.31 ± 0.09	102.0
Leucine	61	9.06 ± 0.11	64.6
Lysine	48	8.33 ± 0.20	123.1
Methionine + Cystine	23	3.85 ± 0.18	118.7
Phenylalanine + Tyrosine	41	10.44 ± 0.27	161.1
Threonine	25	6.24 ± 0.17	177.2
Tryptophan	6.6	1.11 ± 0.09	120.3
Valine	40	5.64 ± 0.21	100.0
Dispensable amino acids			
Alanine	-	5.10 ± 0.30	-
Arginine	-	4.69 ± 0.11	-
Aspartic acid	-	12.74 ± 0.52	-
Glutamic acid	-	11.22 ± 0.55	-
Glycine	-	5.20 ± 0.11	-
Proline	-	5.05 ± 0.19	-
Serine	-	5.06 ± 0.21	-

**Table 3:** Calculated protein efficiency ratio (PER) values of PJPC.

Parameter	PJPC
PER_1_	3.21 ± 0.02
PER_2_	3.13 ± 0.05
PER_3_	1.87 ± 0.02

**Table 4:** Mineral composition of PJPC.

Mineral	Content (mg/100 g)	% PRI/AI
Fe	30.5 ± 4.2	191
Mn	3.79 ± 0.17	126
K	4341 ± 271	124
Cu	1.14 ± 0.07	114
Mg	241 ± 9	80
Ca	118 ± 8	12
Zn	6.04 ± 0.11	60
Na	84.5 ± 4.9	-

**Table 5:** Antioxidant activity expressed as the ferric-reducing antioxidant power (FRAP), Trolox equivalent antioxidant capacity (TEAC), total phenolic compounds (TPCs), and total flavonoid compounds (TFCs).

Parameter	PJPC
FRAP (mmol g^−1^)	1.41 ± 0.07
TEAC (mmol g^−1^)	1.53 ± 0.12
TPC (mg g^−1^)	5.82 ± 0.16
TFC (mg g^−1^)	2.10 ± 0.11

**Table 6:** Cytotoxic doses of PJPC [mg/mL].

Cell Line	IC_10_	IC_50_	IC_90_
Hs 746T	9.11 ± 0.96 ^a^	14.69 ± 0.57 ^a^	23.76 ± 0.65 ^a^
HT-29	0.80 ± 0.14 ^c^	3.89 ± 0.21 ^c^	19.38 ± 2.24 ^b^
CCD 841 CoN	4.71 ± 0.57 ^b^	6.50 ± 0.20 ^b^	9.06 ± 1.19 ^c^

Mean values denoted by different letters (a–c) differ statistically significantly (*p* < 0.05).
